# Caffeine Mitigates Adenosine-Mediated Angiogenic Properties of Choroidal Endothelial Cells Through Antagonism of A_1_ Adenosine Receptor and PI3K-AKT Axis

**DOI:** 10.3390/cells15010087

**Published:** 2026-01-05

**Authors:** SunYoung Park, Yong-Seok Song, Xuan Feng, Christine M. Sorenson, Nader Sheibani

**Affiliations:** 1 Department of Ophthalmology and Visual Sciences, University of Wisconsin School of Medicine and Public Health, Madison, WI 53705, USA; spark67@wisc.edu (S.P.); song224@wisc.edu (Y.-S.S.); xfeng97@wisc.edu (X.F.); 2 McPherson Eye Research Institute, University of Wisconsin School of Medicine and Public Health, Madison, WI 53705, USA; cmsorenson@wisc.edu; 3 Department of Pediatrics, University of Wisconsin School of Medicine and Public Health, Madison, WI 53705, USA; 4 Department of Cell and Regenerative Biology, University of Wisconsin School of Medicine and Public Health, Madison, WI 53705, USA

**Keywords:** age-related macular degeneration, inflammation, purinergic receptors, angiogenesis, cell migration, capillary morphogenesis, choroidal endothelial cells

## Abstract

**Highlights:**

**What are the main findings?**

- Antagonism of the A_1_ adenosine receptor by caffeine is a major mechanism underlying the attenuation of choroidal neovascularization.
- Modulation of intracellular cAMP levels through the engagement of other adenosine receptors also contributes to the anti-angiogenic effects of caffeine.

**What are the implications of the main findings?**

- Targeting the A_1_ adenosine receptor represents a potential therapeutic strategy to limit choroidal neovascularization.
- The concurrent antagonism or modulation of other adenosine receptors may provide additional benefit and enhance the efficacy of approaches aimed at suppressing choroidal neovascularization.

**Abstract:**

Aging reduces the tissue regenerative capacity, promotes chronic inflammation, and contributes to neurodegenerative diseases, including age-related macular degeneration (AMD). AMD is a leading cause of vision loss in older adults and manifests as dry (atrophic) or wet (neovascular) disease. Although dry AMD is more prevalent, neovascular AMD (nAMD) causes the most severe vision impairment and remains a major public health burden. Oxidative stress-mediated inflammation and dysfunction of retinal pigment epithelium (RPE) cells and choriocapillaris drive early AMD. Neovascular AMD is marked by pathologic choroidal neovascularization (CNV), driven largely by dysregulated VEGF signaling. Anti-VEGF therapies are the current standard of care for nAMD but require frequent intravitreal injections, carry procedure-related risks, and are ineffective in a substantial subset of patients, underscoring the need for new therapeutic approaches. Caffeine, a widely consumed and well-tolerated adenosine receptor antagonist, has emerging relevance in vascular regulation and inflammatory signaling. Extracellular ATP and its metabolites, including adenosine, accumulate under stress and act through purinergic receptors to influence angioinflammatory processes. We recently showed that systemic caffeine administration suppressed CNV in vivo, an effect partly reproduced by the adenosine receptor A_2A_ antagonist Istradefylline. Here, we investigated the cell-autonomous effects of caffeine on mouse choroidal endothelial cells, focusing on its role as an adenosine receptor antagonist and its potential to inhibit pathological neovascularization.

## 1. Introduction

Aging contributes to the onset of various neurodegenerative diseases by reducing the tissues’ regenerative capacity and facilitating chronic inflammation [1]. Age-related macular degeneration (AMD) is a major age-associated ocular disorder that can result in significant vision loss [2]. Among individuals aged 50 years and older, the prevalence of AMD is estimated to range from 9.9% to 19.5% for early-stage AMD and from 1.1% to 3.9% for late-stage AMD [3]. AMD presents in two major forms: dry (atrophic) and wet (exudative). Although the dry form is more prevalent, accounting for nearly 90% of AMD cases, wet or neovascular AMD (nAMD) has the most severe impact on vision and quality of life, with a significant economic burden on both individuals and society [4,5,6].

Oxidative stress-induced damage and dysfunction in the retinal pigment epithelium (RPE) cells lead to the subretinal accumulation of drusen, a hallmark of early AMD [7]. The choroid is a vascular connective tissue layer located between the retina and the sclera that functions to supply oxygen and nutrients to the RPE and photoreceptor cells via the choriocapillaris [8]. The impairment of the choriocapillaris, along with the degeneration of the RPE and photoreceptor cells, is a key pathological feature of dry AMD, which could progress to a later stage referred to as geographic atrophy [9]. Wet AMD is characterized by abnormal blood vessel growth in the choroid, leading to vision impairment. Although vascular endothelial growth factor (VEGF) dysregulation has shown a significant impact on the pathogenesis of nAMD, our knowledge regarding the detailed mechanisms involved remains limited.

VEGF is a well-recognized, potent inducer of angiogenesis, and pathological conditions associated with nAMD lead to elevated VEGF levels, resulting in abnormal blood vessel formation [10]. Currently, the intravitreal injection of anti-VEGF agents to prevent leaky neovascularization is the predominant therapeutic approach for the treatment of nAMD [11,12]. However, injection-related adverse effects and the need for frequent administration remain significant challenges in current AMD management [13]. In addition, a significant portion of nAMD patients do not respond to anti-VEGF treatments. Therefore, there is a strong demand for the development of safer, more effective, and convenient treatment options [14,15].

Caffeine is a widely consumed bioactive compound, and its safety has been well established through long-term human consumption and various epidemiological studies in cancer and cardiovascular diseases [16,17]. Caffeine exerts its physiological and pharmacological effects primarily through the antagonism of adenosine receptors (ARs) in the central nervous system (CNS) and in the regulation of vascular function [18,19]. Adenosine is generated through the enzymatic catalysis of extracellular adenosine triphosphate (eATP) by ectoenzymes expressed on the cell membrane. The tissues’ eATP concentration increases under pathological conditions such as inflammation, hypoxia, and oxidative stress [20,21]. In this context, eATP acts as a danger signal or damage-associated molecular pattern [22], with the P2X7 purinergic receptor as the key responder [23].

Adenosine receptors are members of the G-protein-coupled purinergic receptor family and include A_1_, A_2A_, A_2B_, and A_3_. A_1_ and A_3_ ARs primarily exert inhibitory effects, whereas A_2A_ and A_2B_ ARs mediate stimulatory responses [24]. Given their critical roles in physiological metabolism, numerous AR antagonists are being investigated as potential therapeutics for various diseases, including cancer, Parkinson’s disease, and asthma [25]. Caffeine is a non-selective antagonist of ARs, and its binding affinity is relatively low compared to those of more specific AR antagonists [26]. However, its detailed mechanisms of action remain unknown.

We recently demonstrated that the oral administration of caffeine significantly inhibited neovascular growth in a murine model of choroidal neovascularization (CNV) or nAMD [27]. We also showed that these effects of caffeine can be recapitulated, in part, by Istradefylline, an A_2A_ AR antagonist [27]. However, the cell-autonomous activity of caffeine, as well as other AR antagonists, in the choroid, as well as the underlying mechanisms involved, needs investigation. In the current study, the cell-autonomous inhibitory effects of caffeine on CNV and the intracellular signaling pathways impacted were investigated using choroidal endothelial cells (ChEC) isolated from the mouse choroid, with a focus on its role as an AR antagonist.

## 2. Materials and Methods

### 2.1. Ethics Statement

The experiments performed were in accordance with the Association for Research in Vision and Ophthalmology Statement for the Use of Animals in Ophthalmic and Vision Research. The protocols were approved by the Institutional Animal Care and Use Committee of the University of Wisconsin School of Medicine and Public Health (IACUC assurance number: D16-00239). Euthanasia was carried out by CO_2_ asphyxiation according to approved protocols.

### 2.2. Isolation and Culture of Choroidal EC

Choroidal endothelial cells (ChEC) were isolated as previously described by our group [28]. Briefly, eyes from 4-week-old wild-type Immortomice were harvested, and all connective tissue and muscle was removed from the sclera. Under a dissecting microscope in cold Dulbecco’s Modified Eagle’s Medium (DMEM, D5523; Sigma, St. Louis, MO, USA), the anterior segment, lens, vitreous, retina, and optic nerve were sequentially removed, leaving only a tissue layer composed of the retinal pigment epithelium (RPE), choroid, and sclera. The tissues were rinsed with DMEM, minced into small pieces, and digested with collagenase type I (1 mg/mL in serum-free DMEM, LS004194; Worthington, Lakewood, NJ, USA). Following digestion, DMEM containing 10% fetal bovine serum (FBS, 26140-079; Gibco, Grand Island, NY, USA) was added, and cells were pelleted by centrifugation. The cell pellet was resuspended, passed through a sterile 40 µm nylon mesh (Sefar America Inc., Fisher Scientific, Hanover Park, IL, USA), centrifuged, and washed twice with DMEM containing 10% FBS. Cells were then resuspended in DMEM with 10% FBS and incubated with magnetic beads (11035; ThermoFisher, Carlsbad, CA, USA) pre-coated with anti-PECAM-1 antibody (553370; BD Biosciences, San Jose, CA, USA). After washing the magnetic beads with DMEM containing 10% FBS to remove unbound cells, the bound cells were resuspended in EC growth medium (DMEM supplemented with 10% FBS, 2 mM L-glutamine (25030-081; Gibco), 2 mM sodium pyruvate (11360-070; Gibco), 20 mM HEPES (15630-080; Gibco), 1% non-essential amino acids (11140-050; Gibco), 100 μg/mL streptomycin, 100 U/mL penicillin (15140-122; Gibco), 55 U/mL heparin (H3149-250KU; Sigma), and 100 μg/mL endothelial cell growth supplement (E2759; Sigma)) and plated into a single well of a 24-well plate pre-coated with 2 μg/mL human fibronectin (CB-40008; Fisher Scientific). Endothelial cells were cultured in EC growth medium supplemented with murine recombinant interferon-γ (485-MI; R&D Systems, Minneapolis, MN, USA) at 44 U/mL and at 33 °C in a humidified atmosphere with 5% CO_2_. Cells were maintained and propagated on 1% gelatin (G1890; Sigma)-coated 60 mm dishes prepared in phosphate-buffered saline (PBS, D5523; Sigma).

### 2.3. Cell Viability Assays

The cell viability of ChEC was assessed using the MTS assay (3-(4,5-dimethylthiazol-2-yl)-5-(3-carboxymethoxyphenyl)-2-(4-sulfophenyl)-2H-tetrazolium) (G5421; Promega, Madison, WI, USA). Following coating with 1% gelatin in PBS, ChEC (5 × 10^3^ cells/well) were seeded into 96-well plates and cultured in EC growth medium for two days. Prior to treatment with test molecules, cells were incubated with 1 U/mL adenosine deaminase (ADA, A5043; Sigma) for 30 min to remove endogenous adenosine. Caffeine citrate (51754-0500-1; Exela Pharma Sciences, Lenoir, NC, USA), Bz-ATP (2′(3′)-O-(4-benzoylbenzoyl) adenosine -5ʹ -triphosphate, tri(triethylammonium) salt (HY-136254), NECA (5′-N-ethylcarboxamidoadenosine, HY-103173), DPCPX (A_1_ AR antagonist, HY-100937), Istradefylline (A_2A_ AR antagonist, HY-10888), MRS1754 (A_2B_ AR antagonist, HY-14121), MRS1523 (A_3_ AR antagonist, HY-121119), SU5402 (VEGFR2 inhibitor, HY-10407) (Med Chem Express, Monmouth Junction, NJ, USA), LY294002 (PI3K inhibitor, L9908; Sigma), and AZ 11645373 (P2X7 receptor antagonist, 3317; R&D Systems, Minneapolis, MN, USA) were prepared with the desired concentration in EC growth medium. The cells were then treated with the indicated compounds prepared in EC growth medium and incubated for 24 h at 33 °C (0.1 mL/well). Subsequently, MTS solution was added and incubated for 3 h, after which absorbance was measured at 490 nm using a microplate reader (Epoch, BioTek Instruments, Winooski, VT, USA). Cell viability was expressed as a percentage of the untreated control. All samples were prepared in triplicate, and the experiment was repeated twice.

### 2.4. Cell Proliferation

The cell proliferation assay was conducted by plating cells in multiple sets of gelatin-coated 60 mm tissue culture dishes and counting cell numbers every other day in triplicate. Cells (1 × 10^4^) were initially plated, and the growth medium was replaced every other day with freshly prepared medium containing the test compounds for 8 days.

### 2.5. Transwell Migration Assays

The Transwell migration assay was performed as previously described [29]. Briefly, the bottoms of Costar Transwell inserts with an 8 μm pore size (3422; Corning, NY, USA) were coated with fibronectin (2 μg/mL in PBS) overnight at 4 °C, rinsed with PBS, and blocked with 2% BSA in PBS for 1 h at room temperature with gentle rocking. ChEC were trypsinized and resuspended in serum-free EC growth medium, and 1 × 10^5^ cells in 0.1 mL containing the desired test compounds were added to the upper chamber of the Transwell insert. Cells were incubated for 3 h at 37 °C, fixed with 4% paraformaldehyde (PFA) for 10 min at room temperature, and stained with hematoxylin and eosin as previously described [29]. Alternatively, cells were stained with Alexa Fluor^TM^ 488-phalloidin (A12379; ThermoFisher), 1:1000, for 10 min at room temperature to stain the actin cytoskeleton, followed by staining with DAPI (268298; Sigma), 1:2000, for 1 min at room temperature to visualize cellular nuclei. The membranes were mounted onto glass slides, and the number of migrated cells attached to the underside of the membrane was determined by counting 8 high-power fields (×200 magnification).

### 2.6. Capillary Morphogenesis Assays

Tissue culture plates (35 mm) were coated with 0.5 mL of Basement Membrane Matrix (10 mg/mL, HY-K6001; Med Chem Express) and incubated at 37 °C for at least 30 min to allow solidification. Cells were trypsinized, and 2 × 10^5^ cells were resuspended in 2 mL EC growth medium without serum, containing the respective test compounds. The cell suspension was then added to the matrix-coated plates and incubated at 33 °C for 18 h. Images were captured using a Nikon microscope in digital format. For quantitative analysis, the average number of branch points was determined by counting branch points in 10 high-power fields (×100).

### 2.7. Choroid/RPE Ex Vivo Sprouting Angiogenesis

Choroid–RPE tissues were dissected from 3-week-old male and female C57BL/6J mice and prepared as tissue sections according to previously described protocols [30]. Briefly, freshly isolated tissues were cut into 0.5–1 mm pieces, and 7–8 pieces per eye were embedded in Matrigel (10 mg/mL, 356235; BD Biosciences) in 35 mm culture dishes (0.5 mL/dish). After allowing the Matrigel complexes to solidify by incubating them at 37 °C for 30 min, EC growth medium was added. Test compounds were prepared in growth medium and added after 24 h, and the medium was replaced every other day. Images of sprouting from the explant were taken on day 5. For the quantitative assessment of sprouting, the area of outgrowth from the tissue edge was measured using the ImageJ software, version 1.52j (National Institutes of Health, Bethesda, MD, USA; https://imagej.net/ij/ accessed on 15 April 2024).

### 2.8. Reverse Transcription Quantitative PCR Analysis (RT-qPCR)

RNA was isolated from cells cultured in 60 mm tissue culture plates following exposure to the specified experimental conditions. Total RNA was extracted using the RNeasy Mini Kit (74104; Qiagen, Maryland, CA, USA). cDNA was synthesized from 1 μg of total RNA using the RNA to cDNA EcoDry Premix (Double Primed) kit (639549; Takara, San Jose, CA, USA). The cDNA was diluted 10-fold and used as the template for qPCR assays. qPCR was performed on a QuantStudio 3 Real-Time PCR System (ThermoFisher Scientific) using the TB Green Advantage qPCR Premix (639676; Takara). Amplification was carried out in triplicate under the following conditions: 95 °C for 2 min, followed by 40 cycles of 95 °C for 15 s and 60 °C for 40 s, and a dissociation curve step (95 °C for 15 s, 60 °C for 15 s, and 95 °C for 15 s). The linear regression line for DNA quantification was generated based on the relative fluorescent units (RFU) at a threshold fluorescence value (Ct). Expression levels of target genes were quantified by comparing the RFU at the Ct to the standard curve and normalized to the expression of the housekeeping gene 60S ribosomal protein L13α (Rpl13a). The list of primers used is provided in Table 1.

### 2.9. Western Blot Analysis

Cells (4 × 10^5^) were plated onto 60 mm culture dishes and grown to the desired confluence. To collect conditioned medium (CM) for VEGF analysis, ChEC were rinsed once with serum-free EC growth medium and then incubated in EC growth medium containing 1% FBS and the indicated test compounds for 48 h. The collected CM was centrifuged to remove cell debris. Cells were also lysed in 0.1 mL of lysis buffer (142.5 mM KCl, 5 mM MgCl_2_, 10 mM HEPES, 1% NP-40, 1 mM Na_3_VO_4_, 1 mM NaF) and a protease inhibitor cocktail (11836153001; Roche Biochemicals, Mannheim, Germany). To examine the phosphorylation status of target proteins, cells were serum-starved for 24 h and then stimulated with the indicated test compound for 30 min or exposed to the test compounds in EC growth medium for different durations (1 h, 1 day, or 4 days). After incubation, cells were rinsed with cold PBS containing 1 mM Na_3_VO_4_ and lysed with 0.1 mL of lysis buffer. Protein concentrations were measured using the Pierce™ BCA Protein Assay Kit (23227, ThermoFisher), and lysates containing 50 µg of total protein were mixed with an appropriate volume of 6 × SDS sample buffer. Proteins were separated by SDS-PAGE using 4–20% Tris-glycine gels (XP04202; Invitrogen), transferred to nitrocellulose membranes (10600001; Cytiva, Marlborough, MA, USA), and blocked with blocking buffer (TBS containing 0.05% Tween 20 and 5% skim milk). The membranes were incubated overnight at 4 °C with the following primary antibodies diluted 1:1000 in blocking buffer: anti-phospho-AKT (9271), anti-AKT (9272), anti-phospho-ERK (9106), anti-ERK (9102), anti-phospho-p38 (9211), and anti-p38 (9212) from Cell Signaling Technology (Danvers, MA, USA); anti-β-actin (MA5-05739; Invitrogen); and anti-VEGF (sc-7269; Santa Cruz Biotechnology, Dallas, TX, USA). After washing with TBST (TBS with 0.05% Tween 20), the membranes were incubated with appropriate horseradish peroxidase-conjugated secondary antibodies. Signals were developed using the ECL Western Blotting Detection Reagent (RPN2209; Cytiva).

### 2.10. Intracellular cAMP Measurements

The intracellular cAMP levels in ChEC were determined using the cAMP-Glo Assay (V1501; Promega), according to the manufacturer’s instructions. Briefly, cells (3 × 10^3^) were plated in white opaque 96-well plates (3917; Corning) and incubated for 2 days. For the assay, following 10 min incubation with AR antagonists, test compounds were applied for an additional 10 min at 37 °C. Intracellular cAMP levels were then measured using a luminescent microplate reader (SpectraMax i3x, Molecular Devices, San Jose, CA, USA).

### 2.11. Statistical Analysis

Statistical analysis was performed using GraphPad Prism version 8 for Windows (GraphPad Software, La Jolla, CA, USA). The Shapiro–Wilk test was used to assess the normality of distribution of the data. Student’s unpaired *t*-test (two-tailed) was performed for statistical analysis between two groups. One-way analysis of variance (ANOVA) followed by Tukey’s multiple comparisons test was used to determine significant differences between the means of every possible two groups in all experimental groups. The mean ± standard deviation is shown. *p* < 0.05 was considered significant.

## 3. Results

### 3.1. Effects of Bz-ATP, NECA, and Caffeine on ChEC Viability

**Figure 1:**
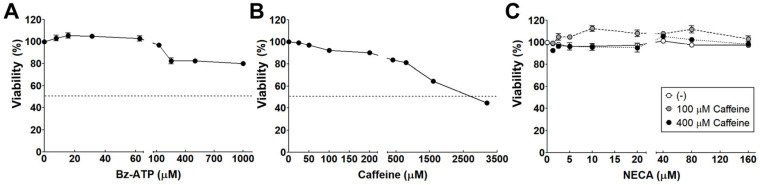
The effects of ATP, adenosine, and caffeine on the viability of ChEC. Cell viability was assessed using the MTS assay following 24 h incubation with varying concentrations of (**A**) Bz-ATP (an ATP analog), (**B**) caffeine, and (**C**) NECA (an adenosine analog) with or without caffeine, as described in the Materials and Methods. Cell viability is presented as a percentage relative to the vehicle control. The dotted lines in (**A**,**B**) indicate the 50% viability mark.

The concentration of eATP increases in various cell types under pathological and stress conditions, including in AMD [31]. Under physiological conditions, the eATP concentration is generally in the nanomolar (nM) range, but its concentration can reach millimolar (mM) levels under pathological and stress conditions [32]. Adenosine is produced from ATP metabolism by enzymes expressed on the cell membrane [33]. To assess the effects of ATP, adenosine, and caffeine on viability, ChEC were incubated with benzoyl ATP (Bz-ATP), a synthetic analog of ATP, for 24 h (Figure 1). ChEC incubated with Bz-ATP at the highest concentration (1000 µM) for 24 h exhibited a 20% decrease in cellular viability. Bz-ATP at less than 100 µM did not show significant changes in ChEC viability (Figure 1A).

Caffeine is an AR antagonist, and its anti-angiogenic effect in choroidal neovascularization was demonstrated in our previous studies [27]. Here, the effect of caffeine on the viability of ChEC was also assessed by incubating ChEC with various concentrations of caffeine for 24 h. Caffeine concentrations higher than 400 µM decreased ChEC viability, reaching an IC_50_ value of 3 mM (Figure 1B). Since adenosine is rapidly metabolized after its production by cellular uptake and/or enzymatic conversion into inosine by adenosine deaminase (ADA) [34], 5′-N-ethylcarboxamidoadenosine (NECA), a high-affinity and metabolically stable AR agonist, was used to investigate the role of adenosine in ChEC viability. As shown in Figure 1C, the adverse effect of NECA on ChEC viability was minimal and co-incubation with 100 or 400 µM caffeine did not alter the impact of NECA on ChEC viability.

### 3.2. Expression of Purinergic Receptors and eATP Metabolizing Enzymes

We also assessed the expression of purinergic receptors (namely adenosine and ATP receptors), enzymes involved in the metabolism of eATP (namely CD39, CD73, ADK, and ADA), and their responses to NECA and/or caffeine in ChEC by RT-qPCR. CD39 is a cell surface enzyme (ectonucleotidase) that breaks down eATP into AMP, while CD73 is the cell surface enzyme converting AMP to adenosine. Adenosine kinase (ADK) controls the cellular level of adenosine by converting it to AMP, while adenosine deaminase (ADA) converts adenosine into inosine. The cell-type-specific expression of these enzymes and their expression changes could significantly impact AR-related functions.

**Figure 2:**
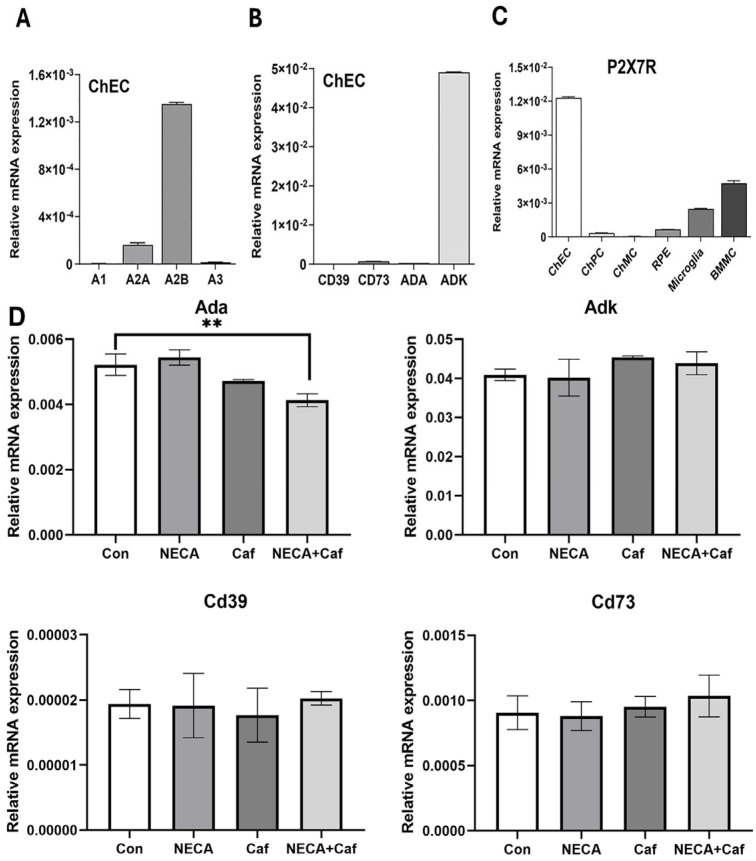
Expression of adenosine and ATP receptors in choroidal cells. (**A**) The expression of ARs in ChEC. Please note the predominant expression of A_2B_ in ChEC. (**B**) Expression of ATP metabolizing enzymes in ChEC. Please note the predominant expression of adenosine kinase (ADK) in ChEC. (**C**) Expression of main ATP receptor P2X7 in various choroidal cells. Choroidal endothelial cells (ChEC), choroidal pericytes (ChPC), choroidal melanocytes (ChMC), retinal pigment epithelium (RPE) cells, microglial cells, and bone marrow-derived mast cells (BMMC) were all generated and characterized in our laboratory from C57BL/6J mice. Please note predominant expression of P2X7 receptor in ChEC. (**D**) Expression of ATP metabolizing enzymes in ChEC incubated with NECA (10 μM), caffeine (Caf, 400 μM), or NECA plus caffeine (NECA + Caf). The impacts of various treatments on the expression of these enzymes were minimal. These experiments were repeated with two additional isolations of these cells (** *p* < 0.01, n = 3).

Figure 2A shows the expression of ARs in ChEC, with the A_2B_ AR being the most prominent. The expression of eATP metabolizing enzymes in ChEC is shown in Figure 2B, with ADK being the most prominent. CD73 was expressed at a 10-fold higher level than CD39, the enzyme involved in generating adenosine. Figure 2C shows that ChEC expressed the highest levels of the P2X7 receptor (dominant ATP receptor) compared to the other choroidal cells examined here. The expression of eATP metabolizing enzymes was not significantly impacted by incubation with NECA, caffeine, or NECA and caffeine, while ADA was downregulated by caffeine alone or with NECA compared to the control (Figure 2D). A similar trend was noted for the expression of adenosine and ATP receptors. Thus, ChEC may favor the presence of ATP (lower expression of CD39) and adenosine (higher expression of CD73), which can be converted to AMP (high expression of ADK).

### 3.3. Caffeine Mitigates Choroid/RPE Ex Vivo Sprouting

**Figure 3:**
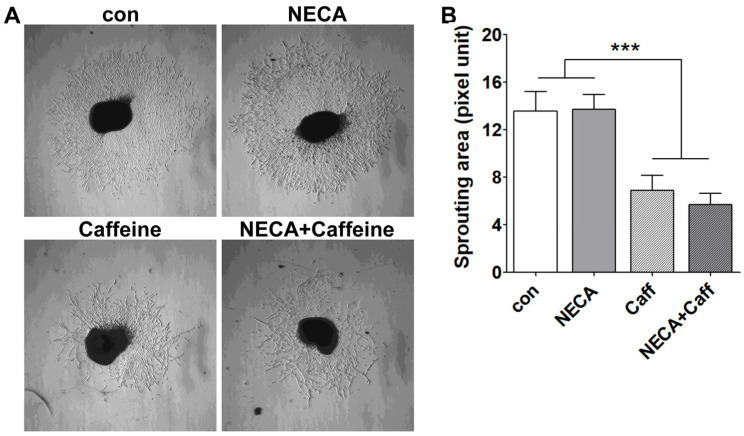
The effects of an AR agonist (NECA) and caffeine on choroid/retinal pigment epithelium ex vivo sprouting angiogenesis. (**A**) Choroid/RPE tissues isolated from 3-week-old C57BL/6J mice were cultured in Matrigel for 5 days in the presence of the vehicle (con), NECA (10 µM), caffeine (400 µM), or a combination of NECA and caffeine. Sprouting outgrowth areas were digitally imaged and quantified using the ImageJ software (**B**). Notably, caffeine treatment significantly reduced sprouting angiogenesis compared to the control and NECA-treated groups (*** *p* < 0.001, n = 3).

Caffeine showed anti-angiogenic effects in ex vivo RPE/choroid explant sprouting assays, and ARs are thought to mediate this inhibition [27]. To explore the effects of NECA on the caffeine-mediated inhibition of ex vivo sprouting, RPE/choroid tissues were incubated with NECA and/or caffeine, and the sprouting area was analyzed. Incubation with NECA showed minimal effects on ex vivo sprouting. In contrast, caffeine significantly suppressed ex vivo sprouting, which was not impacted in the presence of NECA (Figure 3A,B).

### 3.4. NECA Enhances ChEC Proliferation, Which Is Inhibited by Caffeine

Adenosine functions by binding to four G-protein-coupled receptors, namely A_1_, A_2A_, A_2B_, and A_3_ ARs [25]. The expression of ARs in ChEC was analyzed under both normal air conditions (normoxia; 20% O2) and low-oxygen conditions (hypoxia; 2% O_2_). Hypoxia did not lead to significant changes in AR levels. Among the ARs, the A_2B_ AR exhibited the highest expression, followed by the A_2A_ AR, in ChEC, with lower expression of A_1_ and A_3_ ARs under normoxia (Figure 2A and Figure S1).

**Figure 4:**
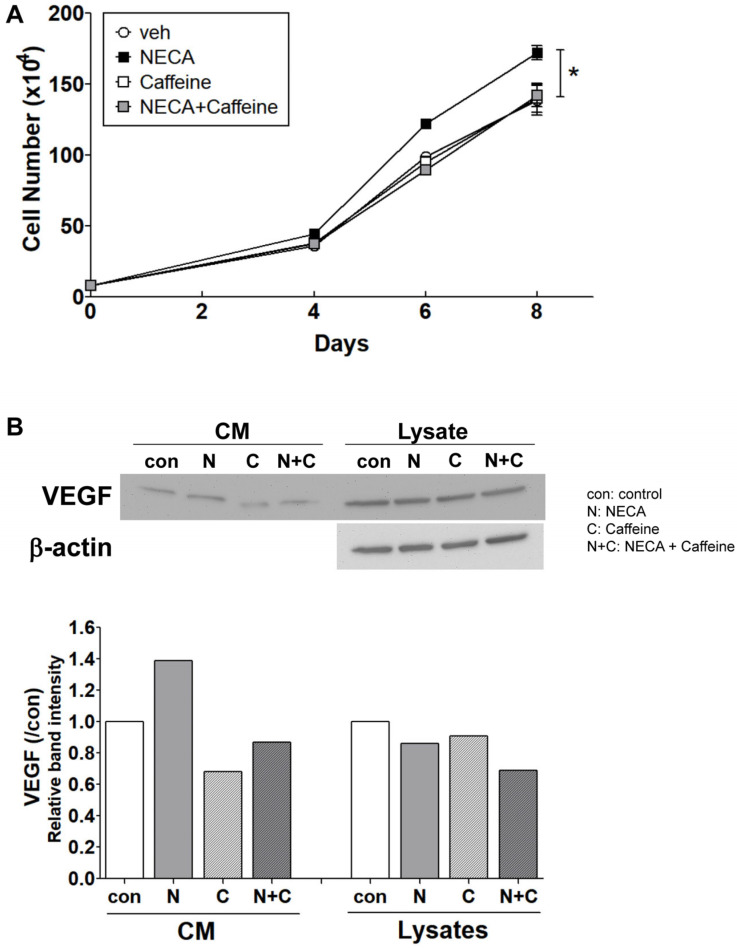
The effects of adenosine (NECA) and caffeine on the proliferation of choroidal endothelial cells (ChEC). (**A**) ChEC proliferation was assessed by counting the number of cells at various time points in the presence of the vehicle (control), NECA (10 μM), caffeine (400 μM), or a combination of NECA and caffeine (* *p* < 0.01; n = 3). (**B**) VEGF production in ChEC was evaluated by Western blot analysis of both conditioned media (CM) and cell lysates after 2-day incubation. Band intensities were assessed using the ImageJ software and are shown in the bottom panel.

Enhanced EC proliferation is one of the essential processes during angiogenesis. The effects of NECA and caffeine on ChEC proliferation were analyzed by counting the number of cells incubated with NECA or NECA and caffeine (Figure 4A). While incubation with 400 µM caffeine did not alter the level of ChEC proliferation, 10 µM NECA enhanced ChEC proliferation, and caffeine inhibited the NECA-mediated enhanced cell proliferation to the level of non-treated control cells (Figure 4A). Vascular endothelial growth factor (VEGF) is a potent inducer of EC proliferation by binding to the VEGF receptor, VEGFR2 [35], expressed on the EC surface. The VEGF production of ChEC was analyzed via the Western blot analysis of a conditioned medium incubated with NECA and/or caffeine. Secreted VEGF levels were increased in NECA-treated cells and decreased in caffeine-treated cells; the NECA-induced increase was mitigated by incubation with caffeine (Figure 4B). The expression of VEGF receptors was not impacted by NECA treatment in ChEC, while the expression of IL-1β was enhanced when ChEC were incubated with NECA (Supplementary Figure S2A,B). Thus, NECA enhanced ChEC proliferation, which was inhibited by caffeine.

### 3.5. Caffeine Mitigated ChEC Capillary Morphogenesis Independently of NECA

**Figure 5:**
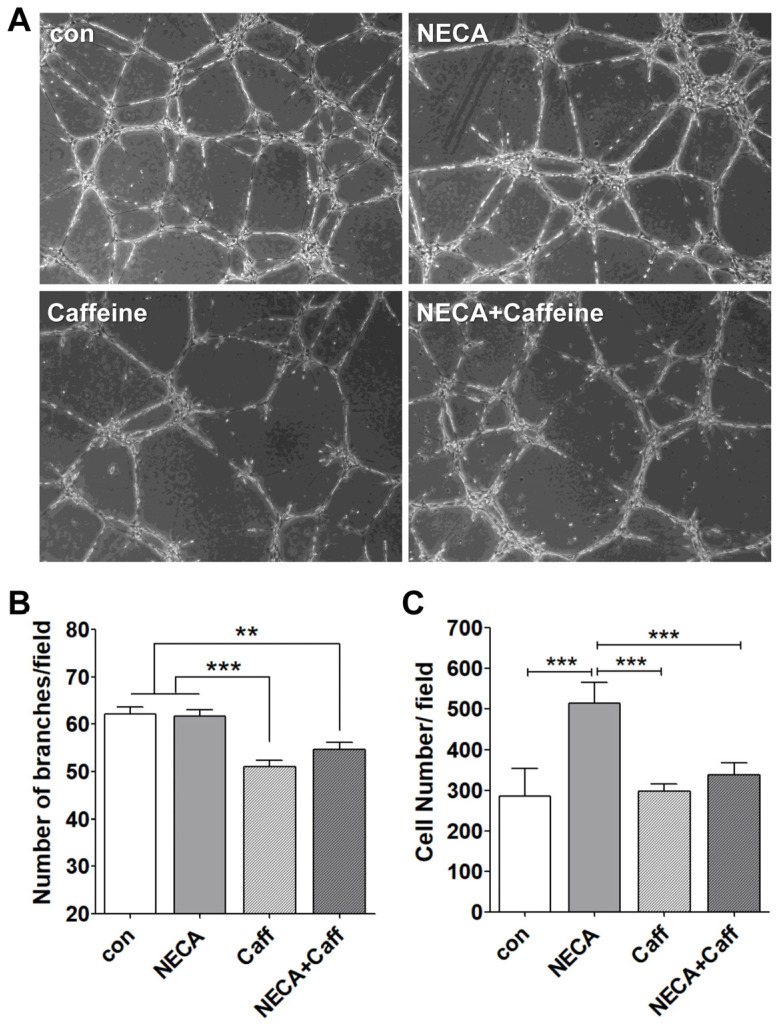
The effects of adenosine (NECA) and/or caffeine on capillary morphogenesis and migration in ChEC. (**A**) ChEC were seeded on Matrigel-coated plates and incubated for 18 h. Tube-like networks were captured in digital images. (**B**) Quantification of capillary morphogenesis was performed by counting the number of branch points in 10 high-power fields (×100; *** *p* < 0.001, ** *p* < 0.01, n = 3). (**C**) Cell migration was assessed using a Transwell assay by counting the number of cells that migrated through the membrane in 8 high-power fields (×200; *** *p* < 0.001, n = 3). “con” is vehicle.

Forming capillary-like structures is a pivotal ability of EC to execute angiogenesis. To investigate the effects of NECA and/or caffeine on capillary morphogenesis, ChEC were plated on Matrigel, and tube formation was analyzed by examining the number of branches formed. Although the incubation of ChEC with 10 µM NECA did not affect capillary morphogenesis, the presence of 400 µM caffeine significantly inhibited this process. This inhibition was not affected by the presence of NECA (Figure 5A,B). These results indicate that caffeine’s disruption of capillary morphogenesis is independent of NECA, consistent with our ex vivo sprouting studies (Figure 3).

### 3.6. NECA Promotes ChEC Migration, Which Is Inhibited by Caffeine

EC migration is a crucial step during angiogenesis when the formation of new blood vessels is demanded. ChEC migration was assessed by analyzing the number of cells migrating through a Transwell filter after incubation with NECA and/or caffeine. While caffeine exerted a minimal effect on ChEC migration by itself, NECA significantly enhanced the ChEC migration that was inhibited by caffeine (Figure 5C). Thus, caffeine by itself does not affect ChEC basal migration, but it inhibits NECA-mediated ChEC migration. Thus, NECA stimulates ChEC proliferation and migration without a significant impact on their capillary morphogenesis. However, caffeine mitigates the proangiogenic activity of ChEC, including capillary morphogenesis.

**Figure 6:**
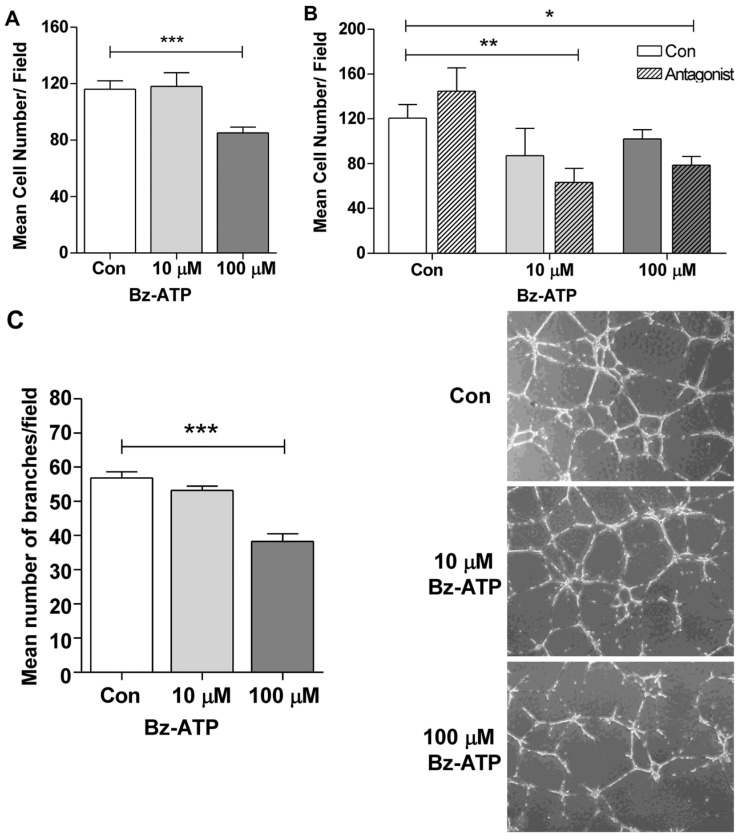
Bz-ATP mitigates ChEC migration and capillary morphogenesis. (**A**) ChEC were incubated with different concentrations of Bz-ATP and their migration was assessed as detailed in the Materials and Methods. In particular, 100 µM Bz-ATP significantly inhibited ChEC migration (*** *p* < 0.001; n = 3). (**B**) The impact of a P2X7 receptor antagonist (AZ 11645373) on ChEC migration with and without Bz-ATP. The antagonist by itself minimally affected ChEC migration. Although the antagonist decreased ChEC migration in the presence of Bz-ATP compared to Bz-ATP alone, it was not significant. (**C**) Bz-ATP (100 µM) significantly mitigated capillary morphogenesis of ChEC in Matrigel (* *p* < 0.05; ** *p* < 0.01; *** *p* < 0.001; n = 3).

ChEC showed significant expression of the main ATP receptor, P2X7, and a lower expression level of CD39 (Figure 2B), the enzyme that breaks down ATP to ADP/AMD. We next assessed the impact of Bz-ATP, an ATP analog and P2X7 agonist, on the proangiogenic properties of ChEC. Figure 6A shows that the incubation of ChEC with Bz-ATP blocked their migration in a concentration-dependent manner. The significant inhibition of migration was noted with 100 µM Bz-ATP, which was further decreased in the presence of the P2X7 receptor-specific antagonist AZ11645373 (Figure 6B). The incubation of ChEC with the antagonist alone had no significant effect on their migration. The incubation of ChEC with Bz-ATP resulted in decreased P2X7 receptor levels, which were restored in the presence of Bz-ATP and its antagonist. The antagonist by itself minimally impacted P2X7 expression. Consistent with the inhibition of ChEC migration, Bz-ATP significantly mitigated the capillary morphogenesis of ChEC (Figure 6C).

### 3.7. Caffeine Mitigates NECA-Mediated AKT Activation, with a Minimal Impact on ERK and P38 MAPK Activation

**Figure 7:**
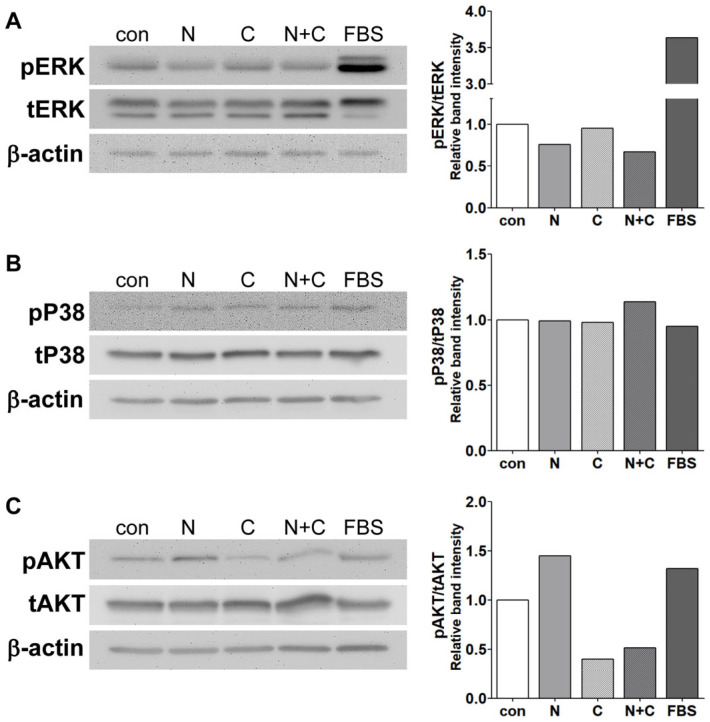
The effects of NECA and/or caffeine on the signaling pathways of ChEC. ChEC were serum-starved for 24 h and then stimulated for 30 min with the vehicle (con), NECA (10 μM), caffeine (400 µM), a combination of NECA and caffeine, or medium containing 10% serum. Activation of ERK (**A**), p38 (**B**), and AKT (**C**) was assessed by Western blot analysis using phosphorylation-specific and total protein antibodies. Band intensities were assessed using the ImageJ software and are shown on the right side of the figure.

To further delineate the downstream signaling pathways involved in the modulation of ChEC’s angiogenic properties, serum-starved (24 h) ChEC were incubated with NECA, caffeine, NECA and caffeine, or serum. The activation of signaling molecules was assessed by Western blot analysis. Serum significantly activated ERK1/2 phosphorylation as expected, whereas NECA modestly attenuated ERK phosphorylation, and caffeine exhibited no effect, while the effect of NECA and caffeine was similar to that of NECA alone (Figure 7A). The activation of P38 MAPK showed no significant increase in ChEC, with the addition of serum following serum starvation. Neither NECA nor caffeine affected P38 MAPK phosphorylation (Figure 7B). However, the incubation of ChEC with NECA resulted in increased AKT phosphorylation, comparable to that induced by serum. In contrast, caffeine significantly inhibited AKT phosphorylation. This inhibition persisted even in the presence of NECA (Figure 7C). Thus, caffeine inhibits the basal and NECA-mediated activation of AKT in ChEC.

### 3.8. Suppression of NECA-Mediated AKT Activation by Caffeine in ChEC Is Maintained Throughout Various Exposure Periods

**Figure 8:**
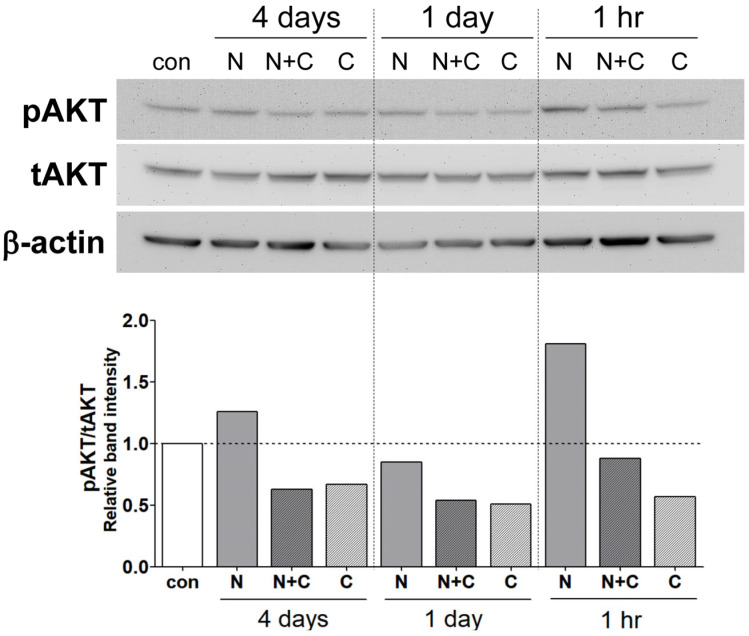
Effects of adenosine (NECA) and caffeine on AKT activation under varying incubation times in choroidal endothelial cells (ChEC). ChEC were cultured in medium containing a vehicle (con), NECA (10 μM), caffeine (400 μM), or a combination of NECA and caffeine for 4 days, 1 day, or 1 h. AKT activation was assessed by Western blot analysis using phosphorylation-specific and total protein antibodies. Band intensities were assessed using the ImageJ software and are shown at the bottom of the figure.

To investigate the effects of caffeine on adenosine-mediated AKT activation, ChEC were exposed to caffeine and/or NECA at various time points, including 1 h, 1 day, and 4 days, under normal growth conditions. ChEC incubated for 1 h showed the most significant activation of AKT by NECA. Exposure for 1 day or 4 days resulted in a modest increase and a decrease in AKT activation compared to the vehicle control, respectively (Figure 8). Caffeine consistently inhibited basal and NECA-mediated AKT activation at all time points, and the effect of NECA on caffeine-mediated inhibition was minimal.

### 3.9. NECA-Mediated Activation of PI3K Results in AKT Activation

**Figure 9:**
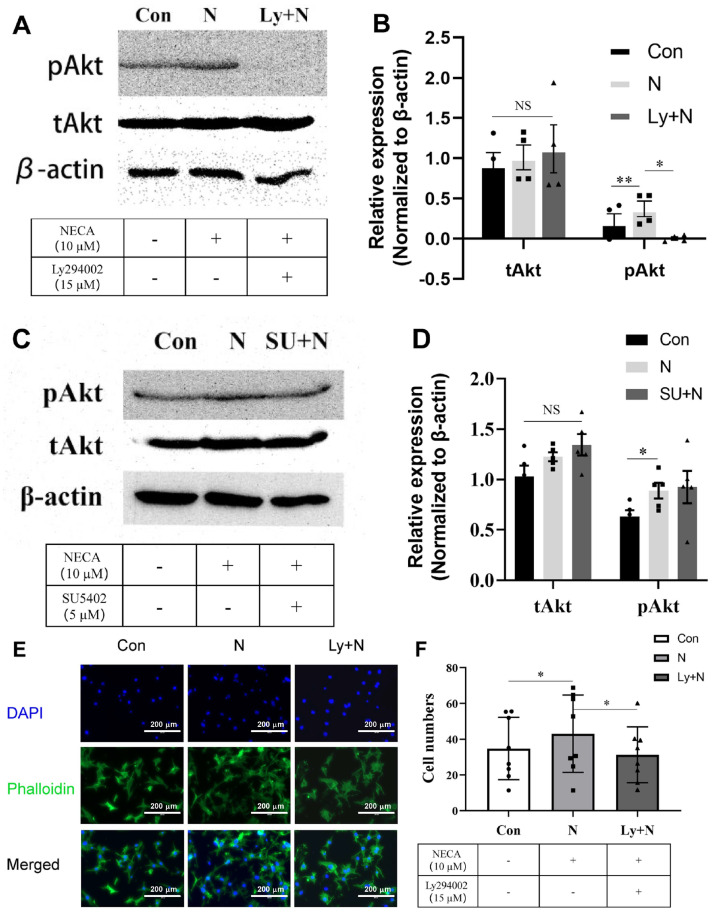
NECA promotes AKT phosphorylation in ChEC, which is dependent on PI3K but not VEGFR2 activity. (**A**). The expression levels of phosphorylated AKT, total AKT, and β-actin were detected by Western blot following incubation with DMSO (con), NECA (N), or LY294002 + NECA (Ly + N) treatment. (**B**) Relative expression of p AKT and t AKT (total AKT) was quantified using ImageJ and Prism software (* *p* < 0.05; ** *p* < 0.01; NS: not significant). NECA-mediated enhanced AKT phosphorylation was independent of VEFGR2 activity. (**C**) The expression of phosphorylated AKT, total AKT, and β-actin was detected by Western blot after DMSO (Con), NECA (N), or SU5402 + NECA (SU + N) treatment. (**D**) Relative expression of p AKT and t AKT was quantified using ImageJ and Prism software (* *p* < 0.05; NS: not significant, n = 5). NECA promotes the migration of ChEC, which is mitigated by incubation with the PI3K inhibitor. (**E**) Phalloidin (green) and DAPI (blue) staining were used to detect migrated ChEC following incubation with DMSO (con), NECA (N), or LY294002 + NECA (Ly + N) within Transwells. (**F**) The cells that were transferred through the Transwell were counted using ImageJ, and data were analyzed using the Prism software (* *p* < 0.05, n = 8).

The engagement of a number of signaling pathways could activate AKT, including PI3K. The interaction of a number of receptor tyrosine kinases, including VEGFR2, with their ligands leads to the activation of PI3K and AKT phosphorylation. To determine whether the activation of PI3K is essential for NECA-mediated AKT phosphorylation, ChEC were incubated with NECA (10 µM), the PI3K inhibitor LY294002 (15 µM), or both, as detailed in the Materials and Methods. Figure 9A,B show that the inhibition of PI3K activity mitigates the NECA-mediated phosphorylation of AKT. Although we noted an increase in VEGF levels with NECA treatment, the inhibition of VEGFR2 using SU5402 (5 µM) had a minimal impact on NECA-mediated AKT phosphorylation (Figure 9C,D). Figure 9E,F show that NECA-mediated AKT phosphorylation enhanced ChEC migration, which was blocked by the PI3K inhibitor. Thus, the activation of PI3K by NECA results in AKT phosphorylation and enhanced cell migration in a VEGF/VEGFR2-independent manner.

### 3.10. Antagonism of ARs and Their Impacts on NECA-Mediated ChEC Migration and AKT Activation

**Figure 10:**
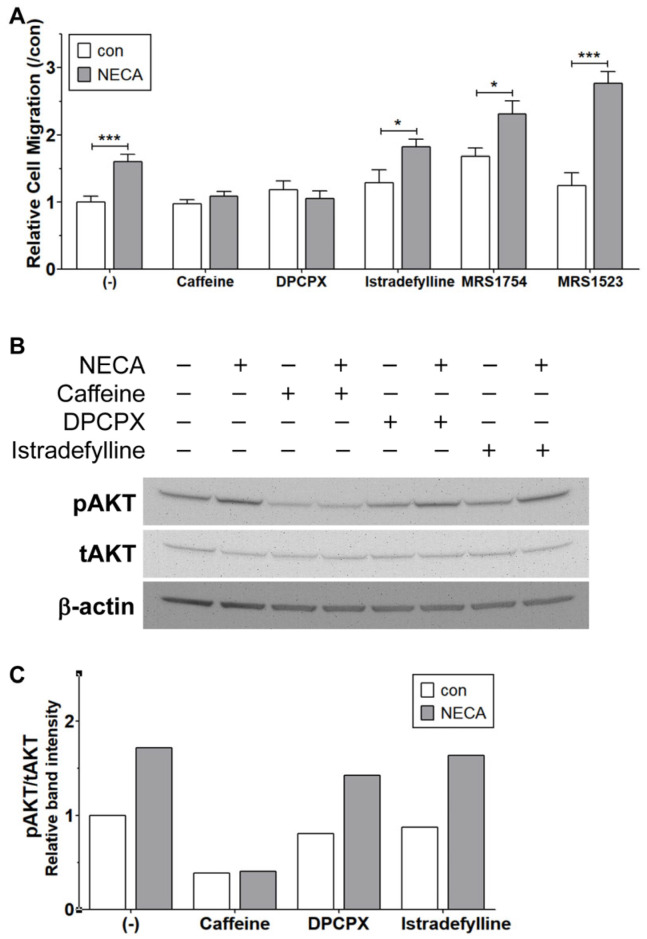
The effects of AR antagonists on ChEC migration and AKT activation. (**A**) Cell migration was assessed using a Transwell assay in the presence of the vehicle (con), NECA (10 µM), or a combination of NECA with caffeine (400 µM), DPCPX (A_1_ AR antagonist, 100 nM), Istradefylline (A_2A_ AR antagonist, 1 µM), MRS1754 (A_2B_ AR antagonist, 100 nM), or MRS1523 (A_3_ AR antagonist, 100 nM). Migration was quantified by counting the number of cells that migrated through the membrane in 8 high-power fields (×200; *** *p* < 0.001, * *p* < 0.05, n = 3). (**B**) AKT activation was determined by Western blot analysis using AKT phosphorylation-specific and total protein antibodies after the incubation of ChEC with the vehicle (con), NECA (10 µM), and combinations of NECA with caffeine, DPCPX (A_1_ AR antagonist, 100 nM), or Istradefylline (A_2A_ AR antagonist, 1 µM) for 1 h. (**C**) Band intensities were assessed using the ImageJ software.

Caffeine is a non-specific AR antagonist, exhibiting a relatively low binding affinity compared to other specific AR antagonists [26]. We next determined which of the ARs were involved in the caffeine-mediated inhibition of ChEC migration in response to NECA. ChEC were incubated with NECA along with various AR antagonists, as detailed in the Materials and Methods. These antagonists included DPCPX (A_1_ AR antagonist), Istradefylline (A_2A_ AR antagonist), MRS1754 (A_2B_ AR antagonist), and MRS1523 (A_3_ AR antagonist). Among the AR antagonists evaluated, DPCPX and caffeine inhibited the NECA-mediated increase in cell migration, while other antagonists had no effect (Figure 10A). Thus, NECA-mediated interaction with A1, but not A_2A,_ A_2B,_ or A_3_ ARs, is essential for the NECA-mediated migration of ChEC. Interestingly, the antagonism of A_2A_, A_2B_, or A_3_ ARs further enhanced NECA-mediated ChEC migration. This could be related to the differences in affinities among these receptors for NECA. However, caffeine, similarly to the A1 AR antagonist, inhibited NECA-mediated ChEC migration.

Next, the effect of DPCPX (A_1_ AR antagonist) on NECA-mediated AKT phosphorylation was assessed by incubating ChEC with NECA or NECA + DPCPX. As Istradefylline (A_2A_ AR antagonist) had no impact on NECA-mediated ChEC migration, its effect on AKT activation was also assessed. Figure 10B,C demonstrate that caffeine inhibited basal and NECA-mediated AKT phosphorylation in ChEC. In contrast, the incubation of ChEC with DPCPX or Istradefylline minimally impacted NECA-mediated AKT phosphorylation. Although the incubation of ChEC with NECA resulted in increased migration and AKT phosphorylation, caffeine mitigated both types of activity. In contrast, DPCPX (A_1_ AR antagonist), like caffeine, inhibited NECA-mediated cell migration without impacting NECA-mediated AKT phosphorylation. However, the incubation of ChEC with Istradefylline had no impact on NECA-mediated migration and AKT phosphorylation. Increased levels of cAMP by NECA or A1 antagonism may drive AKT activation, which is likely influenced by changes in the activity/levels of other ARs and eATP metabolizing enzymes. Caffeine may also impact other intracellular components in addition to A_1_ receptor antagonism, mitigating migration and AKT activation.

### 3.11. Caffeine Modulates NECA-Mediated Proangiogenic Properties of ChEC by Regulating Intracellular cAMP Levels Through A_1_ AR Antagonism

Changes in intracellular cyclic adenosine monophosphate (cAMP), as a second messenger, play vital roles in downstream signaling pathways in various cell types, including endothelial cells [36]. Various enzymes, including adenylyl cyclase (AC) and G-protein-coupled receptors, are involved in cAMP production [37]. Although the engagement of A_1_ and A_3_ adenosine receptors by agonists results in decreased levels of cAMP, the engagement of A_2A_ and A_2B_ results in increased cAMP levels [24]. In addition, A_1_ and A_3_ ARs have higher affinities for adenosine compared to A_2A_ and A_2B_ ARs [24]. Therefore, the expression patterns of different ARs and the concentrations of ligands may contribute to agonistic responses and cAMP levels (Figure 1 and Figure 2).

**Figure 11:**
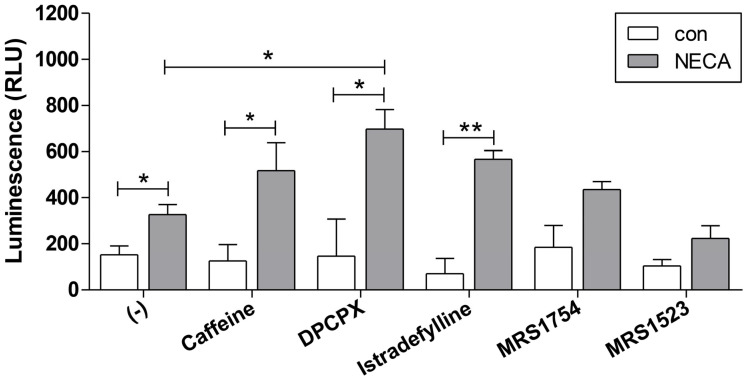
Effects of caffeine and A_1_, A_2A_, A_2B_, and A_3_ AR antagonism on intracellular cAMP production in ChEC. Intracellular cAMP levels were assessed following treatment with the vehicle (con), NECA (10 µM), or a combination of NECA with caffeine (400 µM), DPCPX (100 nM; A_1_ AR antagonist), Istradefylline (A_2A_ AR antagonist; 1 µM), MRS1754 (A_2B_ AR antagonist, 100 nM), or MRS1523 (A_3_ AR antagonist, 100 nM). Antagonists were pre-treated for 10 min, followed by 10 min incubation with NECA (* *p* < 0.05, ** *p* < 0.01; n = 3).

To investigate whether AR antagonists impact the NECA-mediated proangiogenic properties of ChEC through the modulation of intracellular cAMP levels, ChEC were incubated with DPCPX (A_1_ AR antagonist), Istradefylline (A_2A_ AR antagonist), MRS1754 (A_2B_ AR antagonist), MRS1523 (A_3_ AR antagonist), or caffeine (A_1_, A_2A_, and A_2B_ AR antagonist) in the absence or presence of NECA (Figure 11). As expected, the incubation of ChEC with NECA (a non-specific AR agonist) led to an overall increase in intracellular cAMP levels compared to the control, likely through A_2B_ AR agonism (most prominently expressed in ChEC). Incubation with NECA and caffeine resulted in an overall increase in cAMP levels, mostly through A_1_ antagonism (increased cAMP) and A_2B_ (decreased cAMP) ARs, not being significantly different from the increased cAMP seen with NECA alone. This is consistent with the higher affinity of A_1_ ARs for NECA compared to A_2B_ ARs. This notion is further supported by the significant increase in cAMP levels in NECA with the A_1_ AR antagonist compared with NECA alone. Although the A_2A_ AR antagonist with NECA caused an increase in cAMP levels, likely through A_2B_ AR agonism by NECA, this was not significantly different from that seen in NECA alone. The incubation of ChEC with A_2B_ and A_3_ AR antagonists did not significantly increase the cAMP level in the presence of NECA. Thus, the main impact of caffeine is mediated through the antagonism of A_1_ ARs, and its lower level compared to A_1_ AR-specific antagonists likely occurs through the additional antagonism of A_2B_ ARs. The significantly higher levels of cAMP with the A_1_ AR antagonist and NECA compared to caffeine and NECA may explain the inhibition of AKT by caffeine but the lack of AKT inhibition with A_1_ AR and likely A_2A_ AR antagonists (Figure 11).

## 4. Discussion

Caffeine is considered safe by the FDA and is commonly used on a daily basis by many individuals. In humans, moderate caffeine intake (≤400 mg/day or ~6 mg/kg/day) is considered safe, with no consistent evidence of adverse outcomes [38]. Epidemiological studies have also shown that the chronic use of caffeine has long-term neuro- and cardiovascular protective outcomes [39,40,41,42]. The systemic delivery of caffeine, at 10–20 mg/kg during the first two weeks of life, is the standard treatment for neonates born prematurely. This is to prevent apnea, helping with lung development, as well as retinopathy associated with premature birth and exposure to hyperoxia [43]. Although high-dose caffeine (100 mg/kg/day) has been associated with CNS effects in NIH Swiss mice [19], the caffeine treatment dosing is ~10-fold lower. Delineating the molecular and cellular mechanisms by which caffeine modulates AR function represents a significant opportunity to inform its potential therapeutic use in wet AMD.

Wet or exudative AMD involves abnormal vessel growth due to choroidal neovascularization into the retina [12]. Choroidal neovascularization leads to loss of vision, and its inhibition is considered one of the most effective therapeutic strategies [44]. We showed that the oral delivery of caffeine mitigated choroidal neovascularization in a mouse laser model [27]. In the same studies, we showed that, although the specific antagonism of the A_2A_ receptor mitigates neovascularization, it is not as efficacious as caffeine. The formation of new blood vessels requires the activation and functional modulation of endothelial cells, which line the interior surfaces of blood vessels. Thus, understanding the effects of pathological conditions on targeted endothelial cells can provide meaningful insights into the underlying pathophysiological mechanisms. To this end, the study of endothelial cells prepared from the choroid will enhance our understanding of these mechanisms.

The eATP level in patients with AMD is increased as a result of retinal cells responding to cellular damage and inflammation [21,45]. Under physiological conditions, eATP concentrations are typically in the nanomolar range. However, under pathological conditions, the concentration increases to the millimolar range [32]. The incubation of ChEC with Bz-ATP at less than 1 mM showed a 20% reduction in viability (Figure 1). ATP is metabolized into adenosine by cell membrane-associated enzymes, such as CD39 and CD73 [46]. Adenosine then mediates cellular responses through its specific receptors [24]. Adenosine is metabolized by ADA to inosine or ADK to AMP. Therefore, changes in the expression and/or activity of these enzymes could impact the engagement of purinergic signaling pathways modulating various cellular activities. The regulation of angioinflammatory processes is one of the key functions of ARs expressed in endothelial cells. Currently, various AR antagonists are under investigation as potential therapeutic agents for diseases such as cancer [26,47].

Caffeine is a widely consumed bioactive substance that acts as a non-subtype-specific antagonist of ARs, including A_1_, A_2A_, and A_2B_ [48]. Previously, we demonstrated the inhibitory effects of caffeine on CNV in a preclinical model of wet AMD, and the expression of various ARs in the choroid, with the specific antagonism of A_2A_ significantly mitigating CNV [27]. Here, we expanded on this work by delineating the cell-autonomous activity of ChEC in these aspects. To investigate the underlying mechanisms that mediate the inhibitory activity of caffeine on CNV, ChEC were incubated with NECA or Bz-ATP in the presence or absence of caffeine. We assessed the impacts of these treatments on the angiogenic properties of ChEC. The expression levels of A_2A_ and A_2B_ ARs in ChEC were higher than those of A_1_ and A_3_ ARs, and hypoxic conditions or the presence of NECA and Bz-ATP did not alter their expression levels (Figure 2 and Figure S1). However, caffeine reduced cell viability at millimolar concentrations, whereas NECA had no effect on cell viability regardless of the presence of sub-micromolar concentrations of caffeine (Figure 1). Based on these observations, ChEC were incubated with concentrations of test compounds that did not significantly affect cell viability, including 300 µM Bz-ATP, 10 µM NECA, and 400 µM caffeine.

Ex vivo choroid/RPE explant sprouting allowed us to examine how specific molecules affect endothelial cell proliferation and migration [49]. In this assay, NECA exhibited minimal effects on sprouting, whereas caffeine significantly inhibited sprouting. In addition, the inhibitory effect of caffeine was not altered by the presence of NECA (Figure 3). NECA enhanced ChEC proliferation, whereas caffeine suppressed this NECA-induced proliferative effect (Figure 4). Furthermore, NECA induced an increase in VEGF production without altering the expression levels of its receptors. This may function as an autocrine signaling mechanism in ChEC. In angiogenesis, IL-1β promotes endothelial cell proliferation [50,51], and, in the present study, its expression was increased in ChEC following NECA stimulation (Supplementary Figure S2). Although the effect of IL-1β produced by NECA-treated ChEC on their proliferation was not further investigated in the current study, IL-1β may contribute to enhanced AKT activation and ChEC proliferation, which will benefit from further investigation.

In order to develop functional vessels, proliferating endothelial cells must migrate and organize into tube-like structures. While NECA had minimal effects on ChEC capillary morphogenesis, caffeine markedly inhibited this process in the presence or absence of NECA (Figure 5). In addition, NECA enhanced ChEC migration, whereas caffeine counteracted this NECA-induced effect. The inhibitory effects of caffeine on the angiogenic features of ChEC may account for the previously reported reduction in CNV in a caffeine-treated preclinical model [27]. In contrast to NECA, Bz-ATP inhibited ChEC migration and capillary morphogenesis (Figure 6). Thus, high eATP associated with AMD pathogenesis and the lower expression of CD39 in ChEC may have adverse effects on the angiogenic properties of ChEC in wet AMD.

To further investigate the cellular signaling pathways involved in the actions of adenosine and caffeine in ChEC, serum-starved cells were stimulated with NECA in the presence or absence of caffeine. The phosphorylation levels of MAP kinases, including ERK and p38, as well as AKT, were assessed. ERK activation is known to regulate endothelial cell proliferation and migration during angiogenic processes, and the NECA-induced proliferation of ChEC was attenuated by caffeine. However, compared to serum, a potent ERK activator, incubation with NECA and/or caffeine minimally affected ERK phosphorylation (Figure 7).

P38 is considered to play a role in the migration of endothelial cells during angiogenesis [52]. However, despite enhanced ChEC migration by NECA and its inhibition by caffeine, their effects on P38 phosphorylation were minimal. Previous studies have highlighted the crucial role of AKT in EC during angiogenesis by promoting proliferation [53] and migration [54]. NECA treatment resulted in AKT activation in ChEC, which was significantly inhibited by caffeine. Importantly, this caffeine-mediated inhibition was sustained even in the presence of NECA. These results led us to focus on AKT signaling in the adenosine-mediated proangiogenic activation of ChEC and the inhibitory effects of caffeine on these processes. Our studies indicate that caffeine inhibits angiogenic activation in ChEC by modulating AKT signaling and attenuating the proangiogenic effects of adenosine, mainly through the antagonism of A_1_ ARs.

To identify the specific ARs involved in the adenosine-mediated angiogenic activation of ChEC and its inhibition by caffeine, ChEC were incubated with NECA in the presence of selective antagonists for AR subtypes: DPCPX (A_1_ AR antagonist), Istradefylline (A_2A_ AR antagonist), MRS1754 (A_2B_ AR antagonist), and MRS1523 (A_3_ AR antagonist). NECA-mediated ChEC migration was inhibited by caffeine and DPCPX, whereas Istradefylline (A_2A_ AR antagonist), MRS1754 (A_2B_ AR antagonist), and MRS1523 (A_3_ AR antagonist) had minimal effects on NECA-mediated ChEC migration (Figure 10). Thus, the antagonism of A_1_ ARs by caffeine could play a significant role in mitigating NECA-mediated ChEC migration. To further investigate whether the inhibition of ARs affects NECA-mediated AKT activation, ChEC were incubated with DPCPX and Istradefylline in the presence or absence of NECA, and AKT activation was assessed. While caffeine significantly inhibited NECA-mediated AKT activation, neither DPCPX nor Istradefylline affected NECA-mediated AKT activation. Thus, NECA-mediated AKT activation and its inhibition by caffeine may involve the coordinated antagonism of other adenosine receptors, including A_2B_, which is predominantly expressed in ChEC. However, adenosine receptor-independent mechanisms targeted by caffeine, such as the suppression of NECA-mediated IL-1β expression, could be involved. It remains unknown whether NECA-mediated enhanced IL1β production involves adenosine receptor(s).

Adenosine receptors are G-protein-coupled receptors (GPCRs) that modulate cellular responses, primarily through the regulation of cyclic AMP (cAMP) production [24]. Typically, A_1_ and A_3_ AR activation inhibits cAMP production, whereas A_2A_ and A_2B_ AR activation enhances cAMP production in response to adenosine. ChEC demonstrated a significant increase in cAMP production following incubation with NECA. This increase in cAMP was not observed in the presence of A_2B_ and A_3_ AR antagonists (Figure 11). However, a significant increase in cAMP levels in response to NECA was noted in the presence of caffeine and the A_1_ AR antagonist, similar to that seen with NECA alone. Incubation with the A_1_ AR antagonist showed the highest cAMP levels with NECA, compared to caffeine and A_2A_ AR antagonists. These differences in cAMP levels could explain the lack of AKT inhibition with A_1_ and A_2A_ AR antagonists compared to caffeine, likely due to the involvement of multiple ARs engaged by caffeine, including A_1_ and A_2B_ ARs, with lower cAMP levels.

Caffeine represents a promising therapeutic candidate due to its favorable safety profile and ease of administration, as it is a widely consumed bioactive molecule in everyday life. Moreover, our previous studies demonstrated its significant inhibitory effects on neovascularization in the laser-induced CNV model [27]. The increase in eATP concentrations in the choroid in individuals with AMD under pathological conditions led us to investigate the effects of caffeine on neovascularization, considering its role as an AR antagonist. The results of the present study demonstrated that adenosine, substituted by NECA, promoted ChEC proliferation and migration—crucial features of EC during angiogenesis—while caffeine significantly inhibited this angiogenic activation. AKT signaling was markedly responsive to adenosine and increased cAMP levels in ChEC, which was mitigated by caffeine reducing the cAMP level below its threshold level needed for AKT activation, likely due to the antagonism of multiple ARs, including A_1_ and A_2B_ ARs, in ChEC. In addition, other pathways may be involved in AKT activation that could be targeted by caffeine, which will benefit from future investigation.

A_1_ and A_3_ ARs have higher binding affinities for adenosine, followed in order by A_2A_ and A_2B_ ARs [26]. An analysis of AR expression in ChEC revealed that the A_2B_ AR was the most highly expressed, followed by A_2A_, A_3_, and A_1_ ARs. This expression pattern may be considered reasonable in the context of the receptors’ relative binding affinities. A_1_ and A_3_ ARs are involved in cellular signaling by inhibiting cAMP production, whereas A_2A_ and A_2B_ ARs function by stimulating cAMP production [20]. Caffeine is a non-selective AR antagonist and exhibits a relatively low affinity compared to adenosine, NECA, and other AR antagonists [55]. When cells are incubated with NECA and caffeine, NECA is likely bound to ARs with a higher affinity than caffeine. Thus, it is challenging to rule out the possibility that caffeine modulates adenosine signaling through indirect mechanisms rather than solely through direct AR antagonism. Caffeine also functions by inhibiting phosphodiesterases, leading to increased cAMP levels through reduced degradation [56], and by inducing calcium release [57]. We showed that caffeine treatment elevated cAMP production in ChEC with NECA to a level exceeding that induced by NECA alone. Considering that DPCPX is an antagonist of the A_1_ AR, which typically inhibits cAMP production, the inhibition of the A_1_ AR by DPCPX led to the most significant increase in the cAMP levels of ChEC with NECA. The antagonism of other ARs by caffeine, such as A_2B_ ARs, could compromise the increased cAMP levels through the antagonism of the A_1_ AR alone, as noted with DPCPX. How the activation and antagonism of ARs are coordinated to impact ChEC’s angioinflammatory properties to mitigate neovascularization remains of great future interest.

## 5. Conclusions

**Figure 12:**
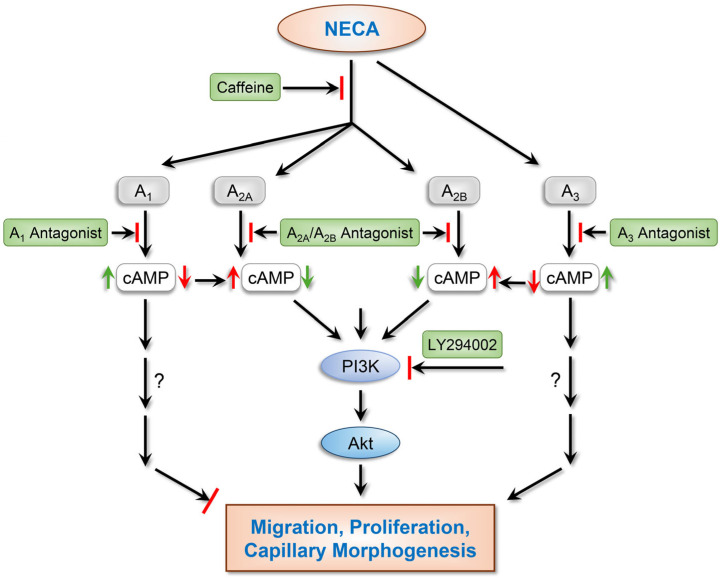
Modulation of angiogenic properties of ChEC by NECA and antagonism of ARs. NECA is an AR agonist acting on A_1_ and A_3_ ARs (higher affinity) by reducing cAMP levels, while its interactions with A_2A_ and A_2B_ ARs (lower affinity) increase cAMP levels. Here, NECA (10 µM) enhanced the proliferation and migration of ChEC, with a minimal impact on their capillary morphogenesis. Caffeine mediates the majority of its action through the antagonism of A_1_, A_2A_, and A_2B_ ARs. The NECA-mediated enhanced migration of ChEC was mitigated by caffeine and A_1_ AR-specific antagonists and not by A_2A_ or A_2B_ AR antagonists. Thus, the caffeine-mediated inhibition of ChEC migration occurs through the antagonism of A_1_ ARs. We found that the activation of AKT by PI3K is essential for the NECA-mediated migration of ChEC, which is mitigated by caffeine. Although A_1_ AR antagonists, like caffeine, inhibited NECA-mediated ChEC migration, they did not affect NECA-mediated AKT activation, perhaps due to high cAMP levels. It is also possible that caffeine may utilize noncanonical pathways to mitigate ChEC migration. Although NECA mediated enhanced cAMP levels, caffeine or AR antagonists failed to mitigate NECA-mediated increased cAMP levels, with the exception of the A_2B_ AR. Delineating the identities of the noncanonical pathways involved and how their coordinated activities impact ChEC’s response to NECA and antagonists of ARs is vital to the specific targeting of these pathways for therapeutic intentions.

In summary, to investigate how caffeine inhibits neovascularization in the mouse CNV model [27], isolated ChEC were incubated with caffeine and analyzed for their angiogenic features. Caffeine primarily functions as an antagonist of ARs and contributes to the regulation of cAMP levels (Figure 12). This is significantly impacted by the levels of agonists and the coordinated engagement and antagonism of various ARs. ChEC incubation with caffeine, with and without NECA, exhibited anti-angiogenic properties, with significant alterations in AKT signaling, likely influenced by the levels of cAMP. NECA enhanced ChEC migration through the activation of the PI3K/AKT axis, likely through the engagement of the A_1_ AR, since only A_1_ AR antagonism, and not A_2A_, A_2B_, or A_3_ ARs, inhibited NECA-mediated ChEC migration. A_1_ AR antagonism also showed the highest levels of cAMP in response to NECA. The A_2A_ AR antagonism minimally impacted the NECA-mediated significant increase in cAMP levels, while A_2B_ AR antagonism blocked the significant increase in NECA-mediated cAMP levels. Caffeine did not negatively impact the NECA-mediated increase in cAMP but caused a modest increase, likely through A_1_ AR antagonism. The A_1_ AR-specific antagonist significantly increased the cAMP level compared to NECA. The reduced impact of caffeine on cAMP levels could be attributed in part to its ability to also antagonize A_2B_ ARs. These differences in cAMP levels may explain the inhibition of NECA-mediated AKT activation by caffeine and not by A_1_ and A_2A_ AR antagonists. Collectively, the current studies provide new insights into how caffeine exerts its inhibitory effects on the angiogenic activity of ChEC in response to adenosine and establishes an important role for cAMP and the PI3K/AKT signaling axis in these activities. Further delineation of the underlying mechanisms responsible for NECA-mediated enhanced AKT activation will benefit from the investigation of canonical and non-canonical signaling pathways involved and the roles that different ARs play in these activities.

## Figures and Tables

**Table 1:** Gene-specific primer sequences.

Protein	Gene	Forward 5′ to 3′	Reverse 5′ to 3′
A_1_ AR	*Adora1*	GTCAAGATCCCTCTCCGGTA	CAAGGGAGAGAATCCAGCAG
A_2A_ AR	*Adora2a*	GGTCCTCACGCAGAGTTCC	TCACCAAGCCATTGTACCG
A_2B_ AR	*Adora2b*	CCGATATCTGGCCATTCG	AGTCAATCCAATGCCAAAGG
A_3_ AR	*Adora3*	CTCTTTGCTAGGATTGCTTGG	AGAAGGAATGCCAAGAGCAG
IL-1β	*Il1b*	GTTCCCATTAGACAACTGCACT	CCGACAGCACGAGGCTTTT
MCP-1	*Ccl2*	GTCTGTGCTGACCCCAAGAAG	TGGTTCCGATCCAGGTTTTTA
TNF-α	*Tnf*	ACCGTCAGCCGATTTGCTAT	TTGACGGCAGAGAGGAGGTT
VEGFR1	*Flt1*	GGCCCGGGATATTTATAAGAAC	CCATCCATTTTAGGGGAAGTC
VEGFR2	*Kdr*	CCCCAAATTCCATTATGACAA	CGGCTCTTTCGCTTACTGTT
RPL13α	*Rpl13a*	TCTCAAGGTTGTTCGGCTGAA	GCCAGACGCCCCAGGTA

## Data Availability

All data used in the studies are presented here.

## Supplementary Materials

The following supporting information can be downloaded at https://www.mdpi.com/article/10.3390/cells15010087/s1. Figure S1: Expression of adenosine receptors in ChEC under normoxia and hypoxia; Figure S2: Impact of NECA on expression of VEGF receptors and inflammatory mediators.
