# Supplementary material for: Caffeine Mitigates Adenosine-Mediated Angiogenic Properties of Choroidal Endothelial Cells Through Antagonism of A1 Adenosine Receptor and PI3K-AKT Axis
Source: Cells. 2026 Jan 5;15(1):87. doi: 10.3390/cells15010087 (PMC12785545; doi:10.3390/cells15010087)
Supplement: Supplementary file 1 [file cells-15-00087-s001.zip › cells-4066281-supplementary.pdf]

Supplementary Materials

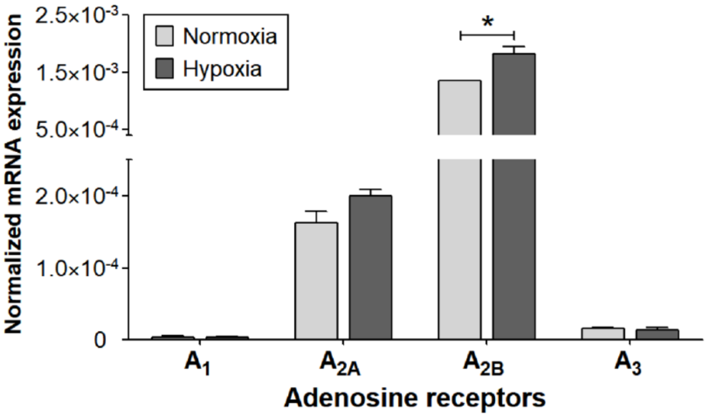

Supplementary Figure S1. Expression of adenosine receptors in choroidal endothelial cells (ChEC). ChECs were cultured under normoxia (20%) or hypoxic conditions (2%) for 24 h, and the expression levels of adenosine receptors were analyzed by reverse transcription quantitative PCR (RT-qPCR) using gene-specific primers. (*P < 0.001; n = 3)

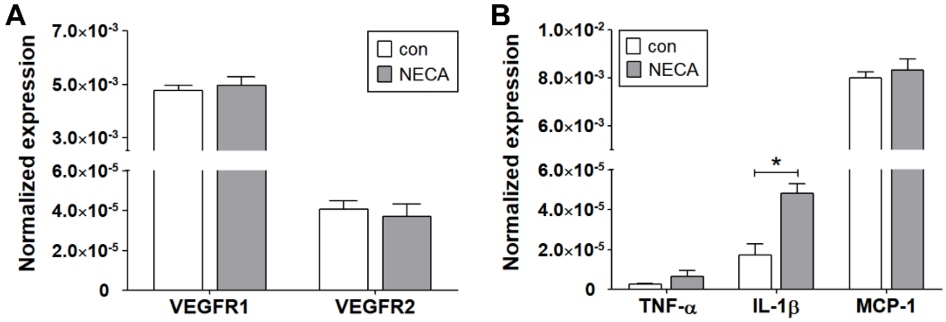

Supplementary Figure S2. Effect of adenosine (NECA) on the expression of VEGF receptors and inflammatory factors in choroidal endothelial cells (ChEC). ChEC were cultured in the presence of NECA (10 mM) for 24 hours, and the expression levels of VEGF receptors (A) and inflammatory factors (B) were analyzed by reverse transcription quantitative PCR (RT-qPCR) using gene-specific primers. (*P < 0.01; n = 3)
